# A multi-authority attribute ring signature supporting dynamic policies and dual anonymity for zero-trust networks

**DOI:** 10.1038/s41598-026-40089-2

**Published:** 2026-02-17

**Authors:** Jinhong Chen, Xueguang Zhou, Wei Fu, Yihuan Mao, Jiaqi Wang

**Affiliations:** 1 Naval University of Engineering, Wuhan, 430033 China; 2 College of Economics and Trade, Jiangxi Vocational College of Finance and Economics, Jiujiang, 332000 China; 3 Jiujiang Shuangfeng Primary School, Jiujiang, 332000 China

**Keywords:** Decentralized identity, Zero trust, Attribute-based signature, Anonymous signature, Multi-authority, Mathematics and computing, Physics

## Abstract

The advent of Decentralized Identity (DID) technology is fundamentally changing the way digital identity is managed, allowing user-controlled, privacy-preserving authentication across trust domains a fundamental requirement if zero trust architectures are to be realized, in which continuous verification and least-privilege access are inherent properties. Under traditional ABS (attribute-based signature) schemes, these are difficult to achieve as fine-grained access control is not always possible in practice and anonymity may not be straightforward when policy is evolving dynamically and different authorities may be involved. In this paper, we present a new multi-authority attribute ring signature scheme, which leverages DID philosophy and anonymous credential techniques, enabling users to mix attributes dynamically according to the policies of veriers without disclosing their pseudonyms or partial attributes. The proposed scheme enables distributed key generation by multiple authorities and is shown to be secure in the random oracle model, achieving existential unforgeability against adaptive chosen-message, identity, and attribute attacks (EUF-CMIAA) as well as full signer and attribute anonymity. Based on the SM9 cryptographic standard, our approach reduces the number of $\mathbb {G}_T$ exponentiations and scalar multiplications during signing by approximately 30% compared to existing ring signatures, offering a practical and efficient authentication solution for emerging DID-driven zero-trust networks.

## Introduction

With the progression of digital transformation in enterprises, the network structures of numerous companies have grown increasingly intricate and are progressively transitioning to the cloud^1,2^. Nevertheless, border-based gateway identity and access control systems struggle to address emerging threats, resulting in escalating security risks^3^. When enterprises employ traditional security paradigms to tackle these challenges, the zero-trust concept offers a novel security perspective^4^. The Jericho Forum introduced the initial iteration of zero trust^5^. Meanwhile, John Kindvag, a former analyst at Forrester Research, officially coined the term “zero trust” and articulated the zero-trust architecture principle with the mantra “never trust, always verify”^6^.This principle mandates that every access request must come with the minimum necessary privileges and verifiable identity or attribute credentials^4^. The rapid emergence of Decentralized Identity (DID) technologies has re-defined digital identity management by shifting control from centralized authorities to individual users^7,8^.Meanwhile DID has aligned perfectly with the identity management and granular access control requirements of zero-trust networks^9,10^.Leveraging distributed ledgers, DID systems enable privacy-preserving, cross-domain authentication without relying on a single trusted third party^11^. Despite this paradigm shift, two intertwined challenges remain open: enforcing fine-grained access control and providing anonymous authentication in a fully decentralized setting.

Recent breakthroughs in succinct non-interactive zero-knowledge proofs (zk-SNARKs) have inspired new privacy-preserving credential systems. Notably, the zk-creds framework^7^ transforms existing identity documents into unlinkable credentials while supporting dynamic policy composition. Yet, these benefits come at the cost of computationally expensive generic zero-knowledge proofs, which limits practical adoption.

Attribute-Based Signatures (ABS) offer an alternative that natively supports fine-grained access control and signer anonymity^12,13^. In an ABS scheme, a signer can create a valid signature only if the attributes embedded in her private key satisfy a predicate specified by the verifier. Although conceptually aligned with DID requirements, traditional ABS constructions suffer from two major limitations: (*i*) policy rigidity caused by linear secret-sharing mechanisms^14–17^, and (*ii*) signature bloat that arises when strong security guarantees are required^18–20^. These drawbacks become critical bottlenecks in real-world deployments involving multiple, mutually distrusting authorities.

**Motivation:** In a zero-trust network, a gateway (verifier) may require a user to prove they hold “Manager” AND “Finance” attributes today, but “Director” OR “Auditor” tomorrow. Traditional ABS requires re-issuing keys for every policy change. Furthermore, standard ring signatures hide the identity but not the attributes. There is a critical lack of a solution that combines **dynamic policy enforcement** (verifier chooses the policy on the fly) with **dual anonymity** (hiding both who signed and which attributes were used) while maintaining **efficiency** suitable for mobile clients.In this paper, we present a new multi-authority attribute ring signature scheme.

### Related work

The evolution of anonymous authentication for zero-trust networks can be categorized into three developmental stages: Traditional Attribute-Based Signatures, Decentralized variants, and SM9-specific adaptations.

#### Traditional and attribute-based signatures (ABS)

The concept of ABS was developed to provide fine-grained access control with signer privacy. Early works, such as Maji et al.^12^ and Guo et al.^15^, established the foundational security requirements. Li and Kim^14^ and Toluee et al.^16^ extended this to attribute-based ring signatures to enhance anonymity, while Li et al.^17^ applied it to personal health records. However, these traditional schemes rely heavily on linear secret-sharing schemes (LSSS) or monotone span programs embedded in the keys.

**Limitation:** This results in “policy rigidity.” While Ling et al.^13^ attempted to achieve dynamic policies, most constructions fix the access structure at issuance. Furthermore, works like Herranz et al.^18^ and Okamoto et al.^19,20^ focused on constant-size signatures but often at the cost of high computational overhead in the standard model.

#### Decentralized and multi-authority schemes

To address the single-point-of-failure in centralized authorities, multi-authority schemes were introduced. Guo et al.^21^proposed a multi-authority ABS resilient to collusion, and Hou et al.^22^ explored designated-combiner signatures. Various functional extensions have also been proposed to address specific needs: Ma et al.^23,24^ introduced blind and designated-verifier ABS for privacy; Zhang et al. proposed Verifier-Policy ABS^25^ and Registered ABS^26^to shift policy control; and others developed puncturable^27^, forward-secure^28^, and proxy signatures^29^ for specific scenarios. Tao et al.^30^ and Kang et al.^31^ focused on lightweight or outsourced designs to reduce client burden.

**Limitation:** Despite these functional rich variants, they often fail to provide “dual anonymity” in a fully distributed setting. They typically hide the identity but leak the attributes, or lack the flexibility to mix attributes from different authorities dynamically. Additionally, recent lattice-based constructions^32–34^ offer post-quantum security but currently suffer from large signature sizes that hinder deployment on constrained devices.

#### SM9-based cryptographic schemes

The SM9 standard^35^, based on bilinear pairings, was designed for high efficiency, with its security formally analyzed by Lai et al.^36^. Recent works have attempted to adapt SM9 for advanced privacy. Tang et al.^37^ and Zhu et al.^38^ proposed traceable and online/offline attribute signatures based on SM9, while Zhou et al.^39^ achieved partial policy hiding.

**Limitation:** While highly efficient, these schemes generally focus on a single authority or lack the ring-signature structure required to hide the signer among a set of potential users completely. Existing SM9 ring signatures, such as the classic ID-based construction by Chow et al.^40^, the standard SM9 scheme by Peng et al.^41^, and the recent work by Xie et al.^42^, achieve identity anonymity but do not natively support dynamic attribute policies. Currently, none of these schemes simultaneously supports multi-authority issuance, dynamic attribute composition, and full anonymity. (see Table 1).

**Table 1:** Comparison of existing functionalities.

Scheme	Multi-authority	Dynamic attributes	Identity anon.	Attribute anon.
Lai et al.^36^	$\times$	$\times$	$\checkmark$	$\checkmark$
Tang et al.^37^	$\times$	$\times$	$\checkmark$	$\checkmark$
Zhu et al.^38^	$\times$	$\times$	$\times$	$\checkmark$

### Our contribution

Leveraging the SM9 signature algorithm and ring signatures, we design the first *multi-authority anonymous attribute ring signature* that supports dynamic attribute composition. Our contributions are as follows:

- **Decentralized key issuance.** Each authority independently issues attribute-specific keys without further coordination.
- **Dynamic policy enforcement.** Signers can combine their attributes on-the-fly to satisfy any access structure chosen by the verifier, without additional interaction with authorities.
- **Dual anonymity.** Both the signer’s identity and the subset of attributes used remain unconditionally anonymous within a ring of potential signers.
- **Provable security.** Under the random oracle model, the scheme is existentially unforgeable against adaptive chosen-message, identity, and attribute attacks (EUF-CMIAA) and achieves full anonymity.
- **Practical efficiency.** Compared with the state-of-the-art SM9 ring signature^42^, our construction reduces $\mathbb {G}_T$ exponentiations and scalar multiplications during signing by approximately 30%, yielding significant performance gains for resource-constrained clients.

## Preliminaries

### Notation

Throughout the paper, we adopt the following conventions.

- $\mathbb {Z}_N^*$ denotes the set $\{1,2,\dots ,N-1\}$; sampling uniformly at random is written $x\!\leftarrow _R\!\mathbb {Z}_N^*$.
- $\{0,1\}^*$ represents the set of all finite-length binary strings.
- *p* and *N* are large primes with $N\mid (p^{12}-1)$ for the 256-bit BN curve used in SM9.
- $\mathbb {F}_p$ is the prime field of order *p*; its extension is denoted $\mathbb {F}_{p^i}$ for $i>1$.
- $E(\mathbb {F}_{p^i})$ is an elliptic curve over $\mathbb {F}_{p^i}$; $\mathbb {G}_1,\mathbb {G}_2\subset E(\mathbb {F}_{p^{12}})$ are cyclic subgroups of prime order *N* with fixed generators $P_1,P_2$ respectively.
- Group law is written additively; scalar multiplication is $[a]P=\underbrace{P+\dots +P}_{a}$.

### Bilinear pairings

Let $(\mathbb {G}_1,+)$ and $(\mathbb {G}_2,+)$ be additive cyclic groups of prime order *N*, and $(\mathbb {G}_T,\cdot )$ a multiplicative cyclic group of the same order. Let $P_1,P_2$ be generators of $\mathbb {G}_1,\mathbb {G}_2$, respectively, and $\psi \!:\mathbb {G}_2\rightarrow \mathbb {G}_1$ an efficiently computable homomorphism such that $\psi (P_2)=P_1$. A (Type-3) bilinear pairing is a map

$$e:\mathbb {G}_1\times \mathbb {G}_2\rightarrow \mathbb {G}_T$$

satisfying

- **Bilinearity:** $e([a]P,[b]Q)=e(P,Q)^{ab}$ for all $P\in \mathbb {G}_1,Q\in \mathbb {G}_2,a,b\in \mathbb {Z}_N^*$.
- **Non-degeneracy:** $\exists \,P\in \mathbb {G}_1,Q\in \mathbb {G}_2$ such that $e(P,Q)\ne 1_{\mathbb {G}_T}$.
- **Efficiency:** *e*(*P*, *Q*) is computable in polynomial time.

Security is based on the hardness of the following problems.

#### Definition 1

*(q-SDH Problem)* Given $(P,Q,[\alpha ]Q,\dots ,[\alpha ^q]Q)$ for unknown $\alpha \in \mathbb {Z}_N^*$, output a pair $(c,[\frac{1}{c+\alpha }]P)$ with $c\in \mathbb {Z}_N$.

#### Definition 2

*(q-BDHI Problem)* Given $(P,Q,[\alpha ]Q,\dots ,[\alpha ^q]Q)$ for unknown $\alpha \in \mathbb {Z}_N^*$, compute $e(P,Q)^{1/\alpha }$.

Both problems are assumed hard in the generic group model and underpin the security of SM9.

### SM9 digital signature scheme

SM9 is an identity-based cryptographic suite standardized by the State Cryptography Administration of China (GM/T 0003-2016). For signatures, it employs a 256-bit Barreto–Naehrig curve with embedding degree $k=12$. Below we summarize the signature component.

#### System setup

A Key Generation Centre (KGC) selects a master secret key $d\!\leftarrow _R\!\mathbb {Z}_N^*$ and publishes the master public key

$$P_{\text {pub-s}}=[d]P_2\in \mathbb {G}_2.$$

#### Private-key extraction

For identity $\textsf{ID}_i$, the KGC computes

$$\textsf{sk}_i=\bigl [d\,(H_1(\textsf{ID}_i)+d)^{-1}\bmod N\bigr ]P_1\in \mathbb {G}_1,$$

where $H_1\!:\{0,1\}^*\rightarrow \mathbb {Z}_N$ is a cryptographic hash.

#### Signature generation

To sign a message *M*, the signer chooses $r\!\leftarrow _R\!\mathbb {Z}_N^*$ and computes

$$w=g^{r},\quad h=H_2(M\Vert w),\quad l=r-h\bmod N,\quad S=[l]\textsf{sk}_i,$$

with $g=e(P_1,P_{\text {pub-s}})\in \mathbb {G}_T$. The signature is $\sigma =(h,S)$.

#### Signature verification

Given $(M,\sigma =(h,S))$, the verifier computes

$$P=[H_1(\textsf{ID}_i)]P_2+P_{\text {pub-s}},\quad u=e(S,P),\quad w'=u\cdot g^{h},$$

and accepts if and only if $h=H_2(M\Vert w')$.

Under the q-BDHI assumption, SM9 signatures are existentially unforgeable against adaptive chosen-message attacks in the random oracle model.

## Methods

### System overview

We consider three distinct entities:

- **Attribute Authorities (AAs):** Trust domains within zero trust networks (such as HR domain, health domain, and finance domain). After collectively generating common parameters, they can independently issue attribute private keys to signers.
- **Signers:** The end-user who possesses a set of attributes and generates ABS. These signatures are created according to the access control structure specified by the verifier.
- **Verifiers:** In a zero trust network, the verifier is typically a zero trust gateway or policy engine. Based on actual circumstances, the verifier generates the corresponding access structure and verifies the ABS produced by the signer in accordance with this structure.

**Figure 1:**
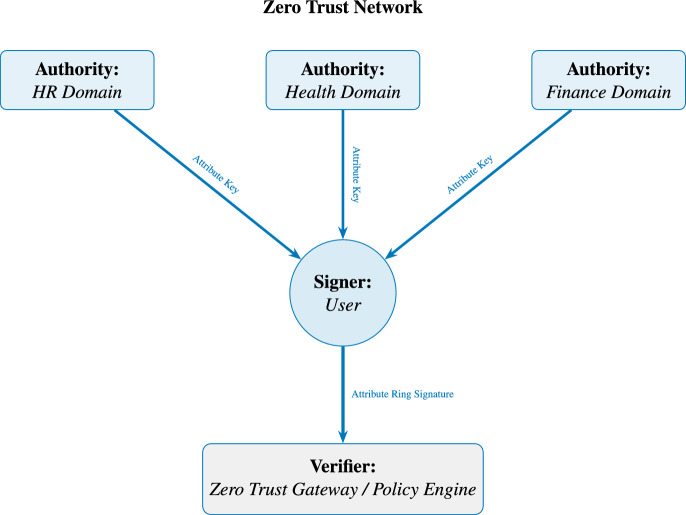
System model.

Figure 1 illustrates the system workflow.

### Formal definition

The scheme consists of four polynomial-time algorithms:

- $\textsf{Setup}(1^\lambda )\rightarrow (\textsf{params},\textsf{mpk})$:A probabilistic algorithm executed jointly by all AAs on input security parameter $\lambda$ to output public parameters $\textsf{params}$ and a master public key $\textsf{mpk}$.
- $\textsf{KeyGen}(\textsf{params},\textsf{mpk},\textsf{ID}_i,\mathscr {A}_{i,j})\rightarrow \textsf{sk}_{i,j}$:A deterministic algorithm run by a single AA to generate an attribute private key $\textsf{sk}_{i,j}$ for identity $\textsf{ID}_i$ and attribute $\mathscr {A}_{i,j}$.
- $\textsf{AttrRingSign}(\textsf{params},M,\mathscr {U},\mathscr {A}_\textsf{c},\pi )\rightarrow \sigma$:A probabilistic algorithm executed by the signer $\pi \in \mathscr {U}$ on message *M*, user set $\mathscr {U}=\{\textsf{ID}_1,\dots ,\textsf{ID}_n\}$, and policy $\mathscr {A}_\textsf{c}$ to produce an attribute ring signature $\sigma$.
- $\textsf{AttrRingVerify}(M,\mathscr {U},\mathscr {A}_\textsf{c},\sigma )\rightarrow \{\texttt{accept},\texttt{reject}\}$:A deterministic algorithm that outputs $\texttt{accept}$ if $\sigma$ is valid and $\texttt{reject}$ otherwise.

#### Correctness

For any honestly generated parameters, keys and signatures,

$$\Pr \!\bigl [\textsf{AttrRingVerify}(M,\mathscr {U},\mathscr {A}_\textsf{c},\sigma )=\texttt{accept}\bigr ]=1.$$

### Security model

We formalise two standard security properties: unforgeability and anonymity.

#### Existential unforgeability under adaptive chosen-message, identity and attribute attacks (EUF-CMAIA)

The EUF-CMAIA game between an adversary $\mathscr {A}$ and a challenger $\mathscr {C}$ proceeds as follows:

- **Initialisation.** $\mathscr {C}$ runs $\textsf{Setup}(1^\lambda )$, gives $\textsf{params}$ to $\mathscr {A}$.
- **Queries.** $\mathscr {A}$ adaptively issues:
  - **Key queries:** $(\textsf{ID}_i,\mathscr {A}_{i,j})$; $\mathscr {C}$ returns $\textsf{sk}_{i,j}$.
  - **Signing queries:** $(M,\mathscr {U}',\mathscr {A}_\textsf{c}')$; $\mathscr {C}$ returns a signature $\sigma$.
- **Forgery.** $\mathscr {A}$ outputs $(M^*,\mathscr {U}^*,\mathscr {A}_\textsf{c}^*,\sigma ^*)$ such that
  - $$\sigma ^*$$ is valid;
  - $\mathscr {A}$ has not queried any key for an identity in $\mathscr {U}^*$ nor any signing query for $(M^*,\mathscr {U}^*,\mathscr {A}_\textsf{c}^*)$.

The advantage of $\mathscr {A}$ is defined as

$$\textbf{Adv}_{\mathscr {A}}^{\text {EUF-CMAIA}}(\lambda )=\Pr [\mathscr {A}\text { wins}].$$

The scheme is EUF-CMAIA secure if for every PPT adversary $\mathscr {A}$ the advantage is negligible in $\lambda$.

#### Full anonymity

Full anonymity requires that neither the signer’s identity nor the subset of attributes used can be linked. The anonymity game is defined between $\mathscr {A}$ and $\mathscr {C}$:

- **Initialisation.** Same as above.
- **Queries.** $\mathscr {A}$ may issue key and signing queries adaptively.
- **Challenge.** $\mathscr {A}$ provides a challenge user set $\mathscr {U}^*$, policy $\mathscr {A}_\textsf{c}^*$, message $M^*$, and two identities $\textsf{ID}_{\pi _0},\textsf{ID}_{\pi _1}$ together with the required attributes. $\mathscr {C}$ flips a bit $b\!\leftarrow _R\!\{0,1\}$ and returns the signature produced by $\textsf{ID}_{\pi _b}$.
- **Guess.** $\mathscr {A}$ outputs a bit $b'$. It wins if $b'=b$.

The advantage is

$$\textbf{Adv}_{\mathscr {A}}^{\text {anon}}(\lambda )=\Bigl |\Pr [b'=b]-\frac{1}{2}\Bigr |.$$

The scheme satisfies full anonymity if $\textbf{Adv}_{\mathscr {A}}^{\text {anon}}(\lambda )$ is negligible for all PPT adversaries.

### Scheme construction

We now present the complete specification of our SM9-based multi-authority anonymous attribute ring signature that supports dynamic attribute composition. All algorithms inherit the pairing groups $(\mathbb {G}_1,\mathbb {G}_2,\mathbb {G}_T,e)$ defined by the SM9 curve.

#### System establishment—$\textsf{Setup}(1^{\lambda })$

All attribute authorities cooperatively execute the following steps:

- Select the public SM9 parameters $(p,N,P_1,P_2,e)$ as described in Section 2.3.
- Each authority *j* chooses an attribute-specific secret $e_j\xleftarrow {\scriptscriptstyle R}\mathbb {Z}_N^*$ for every attribute $\mathscr {A}_j$ under its control and publishes the corresponding attribute public key
  $$P_{\text {pub-}e_j}=[e_j]\,P_2\in \mathbb {G}_2.$$
- The master secret key is implicitly $\textsf{msk}=d\xleftarrow {\scriptscriptstyle R}\mathbb {Z}_N^*$; the master public key is
  $$P_{\text {pub-s}}=[d]\,P_2\in \mathbb {G}_2.$$
- Output
  $$\textsf{params}=\bigl (p,N,P_1,P_2,P_{\text {pub-s}},\{P_{\text {pub-}e_j}\}_{j},e,H_1,H_2\bigr ).$$

#### Attribute private-key generation—$\textsf{KeyGen}(\textsf{params},\textsf{ID}_i,\mathscr {A}_{i,j})$

Given an identity $\textsf{ID}_i$ and an attribute $\mathscr {A}_{i,j}$, the responsible authority computes

$$\textsf{sk}_{i,j}= \frac{e_j}{d+H_1(\textsf{ID}_i\Vert \texttt{hid})} \; P_1\ \in \mathbb {G}_1,$$

where $H_1:\{0,1\}^*\rightarrow \mathbb {Z}_N$ and $\texttt{hid}$ is a public identity-encoding string. The user stores the set $\{\textsf{sk}_{i,j}\}$ locally.

#### Attribute ring signature generation—$\textsf{AttrRingSign}(\textsf{params},M,\mathscr {U},\mathscr {A}_\textsf{c},\pi )$

Let $\mathscr {U}=\{\textsf{ID}_1,\dots ,\textsf{ID}_n\}$ denote the user ring and $\mathscr {A}_\textsf{c}=\{\mathscr {A}_1,\dots ,\mathscr {A}_k\}$ the verifier-specified access policy (attribute conjunction). The signer $\pi \in \mathscr {U}$ proceeds as follows:

- **Eligibility check.** If the attributes held by $\pi$ do not satisfy $\mathscr {A}_\textsf{c}$, abort.
- **Aggregate attribute public keys.** Compute
  $$g_\ell =e\!\Bigl (P_1,\sum _{\mathscr {A}_\ell \in \mathscr {A}_\textsf{c}}P_{\text {pub-}e_\ell }\Bigr ),\quad \ell =1,\dots ,k,$$
  and set $$\textsf{Amp}=\{g_1,\dots ,g_k\}$$.
- **Aggregate private key.** Let
  $$\textsf{sk}_\pi =\sum _{\mathscr {A}_\ell \in \mathscr {A}_\textsf{c}}\textsf{sk}_{\pi ,\ell }\in \mathbb {G}_1$$
  and compute
  $$g_\alpha =e(\textsf{sk}_\pi ,P_2),\qquad g_\beta =e(\textsf{sk}_\pi ,P_{\text {pub-s}}).$$
- **Commit.** Pick random $r,r_0\xleftarrow {\scriptscriptstyle R}\mathbb {Z}_N^*$ and set
  $$w_{\pi ,\xi +1}=g_\xi ^{r_0},\qquad \xi =k.$$
- **Hash chain.** Compute
  $$h_{\pi ,\xi +1}=H_2\!\bigl (\mathscr {U}\Vert \mathscr {A}_\textsf{c}\Vert M\Vert w_{\pi ,\xi +1}\bigr ).$$
- **Ring loop.** For $i=\pi ,\pi +1,\dots ,n,1,\dots ,\pi -1$ (indices modulo *n*) and for $j=\xi +1,\xi +2,\dots ,k,1,\dots ,\xi$ (indices modulo *k*):
  i. If $j>k$ set $j=1$ and $h_{i+1}^1=h_i^{k+1}$; if $i>n$ set $i=1$ and $h_1^1=h_{n+1}^1$.
  ii. $$v_i=H_1(\textsf{ID}_i\Vert \texttt{hid})$$.
  iii. $$\gamma _{i,j}\xleftarrow {\scriptscriptstyle R}\mathbb {Z}_N^*$$.
  iv. $$w_{i,j+1}=(g_\alpha ^{v_i}g_\beta )^{\gamma _{i,j}}\,g_j^{h_{i,j}}$$,      $$h_{i,j+1}=H_2\!\bigl (\mathscr {U}\Vert \mathscr {A}_\textsf{c}\Vert M\Vert w_{i,j+1}\bigr )$$,      $$r_{i,j}=\gamma _{i,j}\,r$$.
  v. If $(i,j)=(\pi ,\xi )$ set $r_{\pi ,\xi }=(r_0-h_{\pi ,\xi })\,r$ and exit both loops.
- **Final response.** Compute
  $$S=[r^{-1}]\,\textsf{sk}_\pi \in \mathbb {G}_1.$$
- **Output.** The signature is
  $$\sigma =\bigl (h_1^1,\,S,\,\{r_{i,j}\}_{i=1..n;\,j=1..k}\bigr ).$$

#### Signature verification—$\textsf{AttrRingVerify}(M,\mathscr {U},\mathscr {A}_\textsf{c},\sigma )$

Upon receiving $\sigma '=(h_1^{\prime 1},\,S',\,\{r_{i,j}'\})$ the verifier proceeds as follows:

- **Pre-compute** $g_\ell =e\!\bigl (P_1,\sum _{\mathscr {A}_\ell \in \mathscr {A}_\textsf{c}}P_{\text {pub-}e_\ell }\bigr )$ for $\ell =1,\dots ,k$.
- **Check formats.** Abort if $h_1^{\prime 1}\notin \mathbb {Z}_N^*$, any $r_{i,j}'\notin \mathbb {Z}_N^*$ or $S'\notin \mathbb {G}_1$.
- **Re-compute chaining values.** Let
  $$g_\alpha '=e(S',P_2),\qquad g_\beta '=e(S',P_{\text {pub-s}}).$$
  For $i=1,\dots ,n$ and $j=1,\dots ,k$ compute
  $$v_i=H_1(\textsf{ID}_i\Vert \texttt{hid}),\qquad w_{i,j+1}'=(g_\alpha ')^{v_i r_{i,j}'}\,(g_\beta ')^{r_{i,j}'}\,g_j^{h_{i,j}'},\qquad h_{i,j+1}'=H_2\!\bigl (\mathscr {U}\Vert \mathscr {A}_\textsf{c}\Vert M\Vert w_{i,j+1}'\bigr ).$$
- **Accept** if and only if $h_1^{\prime 1}=h_{n,k+1}'$.

#### Correctness

Let $\sigma '$ be an honestly generated and un-tampered signature. Then

$$h_1^{\prime 1}=h_1^1,\quad M'=M,\quad S'=S,\quad r_{i,j}'=r_{i,j}\ \forall i,j.$$

The correctness follows from the algebraic derivation presented in the original manuscript, which we reproduce verbatim for completeness.

**Case 1:** For $1\le i<\pi$ or ($i=\pi$ and $1\le j<\xi$)

$$\begin{aligned} w_{i,j+1}&=e\!\bigl ([r^{-1}]\textsf{sk}_\pi ,P_2\bigr )^{\gamma _{i,j}rv_i}\, e\!\bigl ([r^{-1}]\textsf{sk}_\pi ,P_{\text {pub-s}}\bigr )^{\gamma _{i,j}r}\, e\!\Bigl (P_1,\sum P_{\text {pub-}e_j}\Bigr )^{h_{i,j}} \\&=(g_\alpha ')^{v_i r_{i,j}'}\,(g_\beta ')^{r_{i,j}'}\,g_j^{h_{i,j}'} =w_{i,j+1}'. \end{aligned}$$

**Case 2:** For $i=\pi$ and $j=\xi$

$$\begin{aligned} w_{\pi ,\xi +1}'&=e\!\bigl (S',P_2\bigr )^{(r_0-h_{\pi ,\xi })rv_\pi }\, e\!\bigl (S',P_{\text {pub-s}}\bigr )^{(r_0-h_{\pi ,\xi })r}\, e\!\Bigl (P_1,\sum P_{\text {pub-}e_\xi }\Bigr )^{h_{\pi ,\xi }} \\&=e\!\bigl ((r_0-h_{\pi ,\xi })\textsf{sk}_\pi ,v_\pi P_2\bigr )\, e\!\bigl ((r_0-h_{\pi ,\xi })\textsf{sk}_\pi ,P_{\text {pub-s}}\bigr )\, e\!\Bigl (P_1,\sum P_{\text {pub-}e_\xi }\Bigr )^{h_{\pi ,\xi }} \\&=e\!\bigl ((r_0-h_{\pi ,\xi })e_\xi P_1,P_2\bigr )\, e\!\bigl (h_{\pi ,\xi }e_\xi P_1,P_2\bigr ) \\&=e\!\bigl (r_0 e_\xi P_1,P_2\bigr )=g_\xi ^{r_0}=w_{\pi ,\xi +1}. \end{aligned}$$

**Case 3:** For $(i=\pi ,\,\xi <j\le k)$ or $(\pi <i\le n)$, the same algebraic chain ensures $h_{i,j+1}'=h_{i,j+1}$. Since $h_1^1=h_{n,k+1}$ holds, the verification algorithm always returns $\texttt{accept}$.

### Security analysis

We provide formal proofs that the proposed attribute ring signature satisfies *existential unforgeability* (EUF-CMIAA) and *full anonymity* under the q-SDH assumption in the random oracle model. All equations and derivations are kept exactly as in the original manuscript, only refined for clarity and English readability.

#### Unforgeability

##### Theorem 1

Under the random oracle model, if the q-Strong Diffie–Hellman (q-SDH) problem is hard, the proposed attribute ring signature achieves existential unforgeability against adaptive chosen-message, identity and attribute attacks (EUF-CMIAA).

##### Proof

Assume there exists a probabilistic polynomial-time (PPT) adversary $\mathscr {A}$ that wins the EUF-CMIAA game with non-negligible advantage $\varepsilon$. We construct a simulator $\mathscr {S}$ that, given a q-SDH instance

$$\bigl (P,\,Q,\,[\alpha ]Q,\,[\alpha ^{2}]Q,\dots ,[\alpha ^{q}]Q\bigr ),$$

uses $\mathscr {A}$ to output a valid q-SDH solution $(c,[\tfrac{1}{c+\alpha }]P)$.

**Initialisation.** $$\mathscr {S}$$ fixes

- a maximum identity universe $\mathscr {U}_\theta =\{\textsf{ID}_1,\dots ,\textsf{ID}_\theta \}$,
- a challenge identity set $\mathscr {U}^*=\{\textsf{ID}^*_1,\dots ,\textsf{ID}^*_n\}$,
- a maximum attribute universe $\mathscr {A}_\rho =\{\mathscr {A}_1,\dots ,\mathscr {A}_\rho \}$,
- a challenge attribute set $\mathscr {A}^*=\{\mathscr {A}^*_1,\dots ,\mathscr {A}^*_k\}$.

$\mathscr {S}$ chooses $q-1$ distinct values $v_1,\dots ,v_{q-1}\xleftarrow {\scriptscriptstyle R}\mathbb {Z}_N^*$ and sets

$$f(x)=\prod _{i=1}^{q-1}(x+v_i)=\sum _{i=0}^{q-1}c_ix^i.$$

It then computes

$$P_2=[f(\alpha )]Q,\qquad P_1=\psi \!\bigl ([f(\alpha )]Q\bigr ),\qquad P_{\text {pub-s}}=\sum _{i=0}^{q-1}c_i[\alpha ^{i+1}]Q.$$

All public parameters are thus simulated from the q-SDH instance.

**Oracle Simulation.** $\mathscr {S}$ maintains two initially empty lists $\mathscr {L}_1,\mathscr {L}_2$ for $H_1$ and $H_2$.

- $H_1$ **queries.** On input $\textsf{ID}_i$:
  1. If $\textsf{ID}_i\in \mathscr {U}^*$, $\mathscr {S}$ picks $x_i\xleftarrow {\scriptscriptstyle R}\mathbb {Z}_N^*$ and records $(\textsf{ID}_i,x_i)$.
  2. Otherwise, $\mathscr {S}$ assigns the smallest unused $v_l$ to $x_i$, increments *l*, and records $(\textsf{ID}_i,x_i)$.
- $H_2$ **queries.** On input $(\mathscr {U},\mathscr {A}_\textsf{c},M,w)$, $\mathscr {S}$ returns a fresh random value and stores the tuple.
- **Key queries.** On $(\textsf{ID}_i,\mathscr {A}_j)$:
  1. If $$\textsf{ID}_i\notin \mathscr {U}^*$$, $$\mathscr {S}$$ aborts.
  2. Otherwise, $\mathscr {S}$ retrieves $x_i$ and computes
    $$\textsf{sk}_{i,j}=\frac{e_j}{\alpha +x_i}P_1$$
    via polynomial interpolation, which is possible because *f*(*x*) is known.
- **Signing queries.** For queries $(\mathscr {U}',\mathscr {A}_\textsf{c}',M)$ with $\mathscr {U}'\cap \mathscr {U}^*=\varnothing$ and $\mathscr {A}_\textsf{c}'\cap \mathscr {A}^*=\varnothing$, $\mathscr {S}$ simulates a signature by choosing random $S\in \mathbb {G}_1$ and random $\{r_{i,j}\}$ and programming the random oracle accordingly.
- **Forgery and Extraction.** By the Forking Lemma, $\mathscr {S}$ can obtain two valid signatures
  $$\sigma _1=(h_{1,1}^{(1)},S^*,\{r_{i,j}^{(1)}\}),\qquad \sigma _2=(h_{1,1}^{(2)},S^*,\{r_{i,j}^{(2)}\})$$
  on the same $(\mathscr {U}^*,\mathscr {A}^*,M^*)$ such that $h_{\pi ,\xi }^{(1)}\ne h_{\pi ,\xi }^{(2)}$ and $r_{\pi ,\xi }^{(1)}\ne r_{\pi ,\xi }^{(2)}$. Solving the resulting linear equation yields
  $$(v_\pi ,W)=\Bigl (v_\pi ,\tfrac{1}{d}\Bigl (\tfrac{1}{e_\xi }\tfrac{r_{\pi ,\xi }^{(1)}-r_{\pi ,\xi }^{(2)}}{h_{\pi ,\xi }^{(2)}-h_{\pi ,\xi }^{(1)}}S^*-F(\alpha )P\Bigr )\Bigr ),$$
  which satisfies
  $$W=\frac{1}{\alpha +v_\pi }P.$$
  Hence $(v_\pi ,W)$ is a valid q-SDH solution. The success probability is
  $$\frac{(\theta -n)\varepsilon }{n k}\cdot \frac{\varepsilon }{2 q_H}-\frac{q_S}{N},$$
  which is non-negligible whenever $\varepsilon$ is non-negligible.

$$\square$$

#### Anonymity

##### Theorem 2

If the random values used in $\textsf{AttrRingSign}$ are uniformly distributed, the scheme achieves full anonymity: an adversary cannot distinguish the signer’s identity or the subset of attributes actually used beyond the required policy.

##### Proof

Let $\sigma =(h_1^1,S,r_{1,1},\dots ,r_{k,n})$ be a signature generated by user $\pi _1$ holding attributes $\Sigma \mathscr {A}_{\xi _1}\subseteq \mathscr {A}_\textsf{c}$. We show that $\sigma$ can be identically simulated by any other user $\pi _2\in \mathscr {U}$ holding $\Sigma \mathscr {A}_{\xi _2}\subseteq \mathscr {A}_\textsf{c}$.

Observe that

$$S=\frac{\sum e_{\xi _1}}{r(d+v_{\pi _1})}P_1=\frac{\sum e_{\xi _2}}{r'(d+v_{\pi _2})}P_1,$$

where

$$r'=r\cdot \frac{d+v_{\pi _2}}{d+v_{\pi _1}}\cdot \frac{\sum e_{\xi _1}}{\sum e_{\xi _2}}.$$

Since *r* and all intermediate random values $\{r_{i,j}\}$ are chosen uniformly and independently, the joint distribution of $(h_1^1,S,\{r_{i,j}\})$ is identical regardless of which $\pi \in \mathscr {U}$ is chosen. Consequently, any polynomial-time adversary has negligible advantage in distinguishing the real signer or the exact attributes used. $\square$

## Performance evaluation

We provide a comprehensive assessment of both theoretical complexity and empirical performance. All benchmarks are conducted on a Windows 10 workstation equipped with an AMD Ryzen 5 4600H (3.0 GHz, 6 cores, 16 GB RAM). The implementation is written in Python 3.10 on top of the open-source hggm library ^43^.

### Analytical comparison

Table 2 summarises the dominant cryptographic operations for each phase, where

- $T_{\text {sm}_1}$: scalar multiplication in $\mathbb {G}_1$,
- $T_{\text {sm}_2}$: scalar multiplication in $\mathbb {G}_2$,
- $T_{\text {sm}_3}$: scalar multiplication in $\mathbb {G}_T$,
- $$T_{\text {bp}}$$: bilinear pairing,
- $$T_{\text {e}}$$: exponentiation in $$\mathbb {G}_T$$,
- $T_{\text {htp}}$: hash-to-point on the elliptic curve.

**Table 2:** Asymptotic complexity comparison.

Scheme	KeyGen	Sign	Verify
Chow et al.^40^	$T_{\text {htp}}$	$nT_{\text {htp}}+2nT_{\text {sm}_2}$	$nT_{\text {sm}_2}+2T_{\text {bp}}$
Peng et al.^41^	$T_{\text {sm}_1}$	$(n+1)T_{\text {sm}_1}+(n-1)T_{\text {sm}_2}+(n-1)T_{\text {e}}+nT_{\text {bp}}$	$nT_{\text {sm}_2}+nT_{\text {e}}+nT_{\text {bp}}+nT_{\text {sm}_3}$
Xie et al.^42^	$T_{\text {sm}_1}$	$T_{\text {sm}_1}+(3n-2)T_{\text {e}}+2nT_{\text {sm}_3}$	$3nT_{\text {e}}+2T_{\text {bp}}+2nT_{\text {sm}_3}$
Our scheme	$T_{\text {sm}_1}$	$T_{\text {sm}_1}+(2n-1)T_{\text {e}}+nT_{\text {sm}_3}$	$3nT_{\text {e}}+2T_{\text {bp}}+2nT_{\text {sm}_3}$

To evaluate the proposed scheme against the most current standards, we compare our method with **Peng et al.**^41^, which represents the typical SM9 construction, and **Xie et al.**^42^, a recently published (2025) state-of-the-art optimization for SM9 ring signatures. As shown in Table 2, compared with the latest SOTA^42^, our proposed scheme eliminates one fixed-base exponentiation in $\mathbb {G}_T$ and reduces $\mathbb {G}_T$ scalar multiplications by approximately 30%.

### Experimental results

#### Setup and methodology

We instantiate the proposed scheme, Peng et al.^41^, and Xie et al.^42^ in Python using the hggm library ^43^. Each measurement is the mean of 50 independent executions.

#### Latency measurements

Table 3 reports the average running time (ms) for ring sizes $n\in \{4,16,64,256,1024\}$.The results substantiate the theoretical efficiency gains derived in the previous section.

**Table 3:** Measured running time (ms).

Scheme	Phase	4	16	64	256	1024
Peng et al.^41^	Sign	109.46	413.08	1680.76	6720.69	27318.90
Xie et al.^42^	Sign	46.17	188.98	761.19	3034.18	12391.90
**Our Scheme**	Sign	**28.75**	**122.75**	**508.64**	**2037.94**	**8087.21**
Peng et al.^41^	Verify	114.07	408.46	1641.07	6519.69	26419.37
Xie et al.^42^	Verify	196.82	303.20	874.67	3179.10	12463.80
**Our Scheme**	Verify	160.40	332.13	882.94	3151.35	12264.77

**Figure 2:**
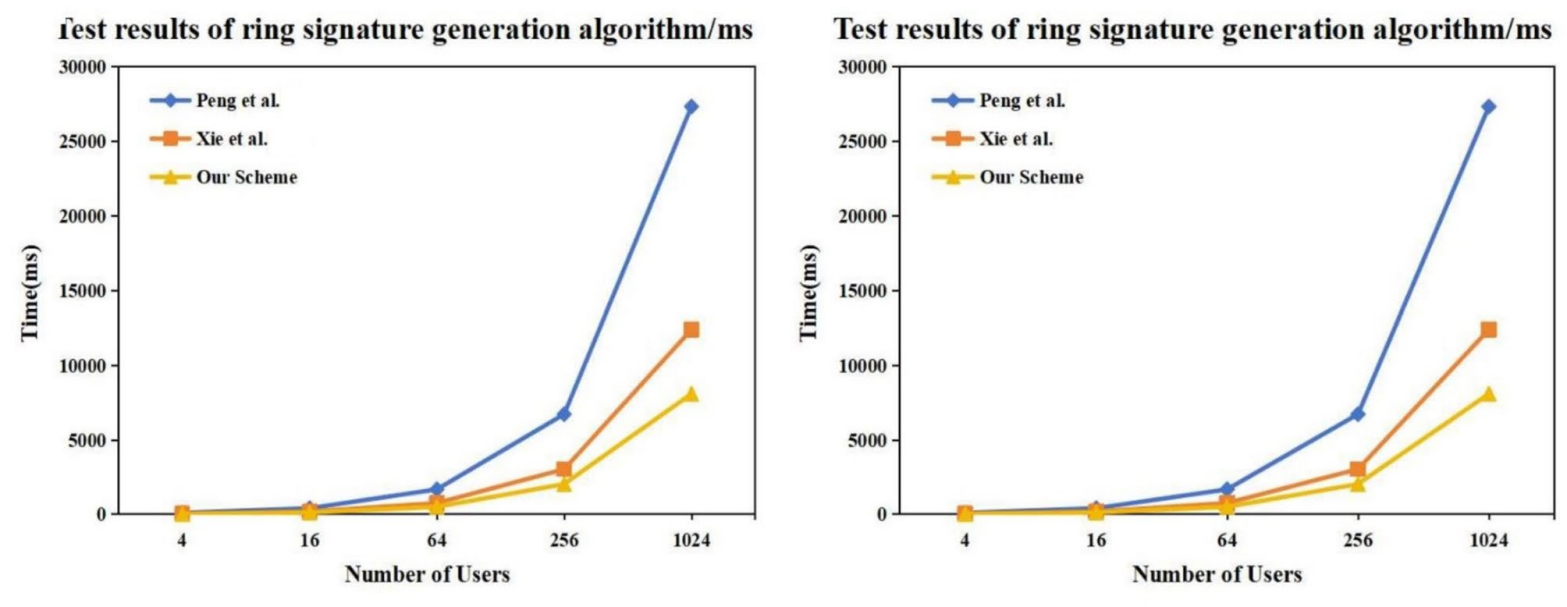
Relative speed-up of the proposed scheme over Peng et al.^41^ and Xie et al.^42^.

**Signature Generation (Client-Side Efficiency):** As illustrated in Fig. 2, the signature generation time for all three schemes grows linearly with the ring size *n*. However, the growth rate (slope) of the proposed scheme is significantly lower than that of the baselines.

- **Comparison with Standard Scheme (Peng et al.**^41^**):** Our scheme achieves a consistent speedup. At $n=1024$, our generation time is 8.08s compared to 27.31s, resulting in a speedup factor of approximately **3.43**x.

- **Comparison with SOTA (Xie et al.**^42^**):** Even against the most recent optimized scheme published in 2025, our approach maintains a clear advantage. Xie et al. requires 12.39s for $n=1024$, whereas our scheme requires only 8.08s, yielding a speedup of **1.53**x.

**Underlying Cause of Improvement:** This performance gap is directly attributable to the algebraic optimizations detailed in Table 2. Operations in the multiplicative group $\mathbb {G}_T$ are significantly more expensive than those in the additive group $\mathbb {G}_1$. By eliminating one fixed-base exponentiation in $\mathbb {G}_T$ and reducing the coefficient of $\mathbb {G}_T$ scalar multiplications from 2*n* (in Xie et al.) to *n* (in our scheme), the computational burden increases much more slowly as the ring size expands. This makes the proposed scheme particularly suitable for resource-constrained devices (e.g., mobile phones or IoT sensors) in decentralized identity systems. versus the most recent optimized scheme by Xie et al.^42^ when $n=1024$.This confirms that our scheme outperforms both the standard implementation^41^ and the latest published optimization^42^.

**Verification (Server-Side Efficiency):** Regarding verification (Table 3 , bottom rows), our scheme performs comparably to the baselines. For $n=1024$, our verification time (12.26s) is almost identical to Xie et al. (12.46s).

This behavior is expected because verification in SM9-based ring signatures is dominated by bilinear pairing operations ($T_{\text {bp}}$) and the reconstruction of the pairing product chain, which are structurally similar across all valid constructions. In a Zero-Trust architecture, verification is typically performed by high-performance policy engines or gateways rather than end-users. Therefore, maintaining standard verification costs while significantly reducing client-side signing latency represents an optimal trade-off for real-world deployment.

**Conclusion of Experiments:** The empirical data confirms that while retaining the strong security properties of SM9, the proposed scheme successfully mitigates the “signature bloat” issue common in ring signatures. The scalability trends in Fig. 2 demonstrate that as the network size (ring size) increases, the efficiency advantage of our scheme becomes increasingly pronounced.

## Discussion

The empirical speed-up is attributed to aggressive pre-computation of fixed-base exponentiations in $\mathbb {G}_T$ and a reduced scalar multiplication count. Since signers are typically resource-constrained clients whereas verifiers are servers, the improvement in signing latency offers practical value. Future work will explore constant-size signatures independent of ring cardinality.

## Conclusion

In this paper, we designed a multi-authority attribute ring signature scheme with the dynamic attribute composition and dual anonymity, which are very useful to satisfy important authentication needs for the zero trust networks based on DID systems. Under the framework of SM9 and with combination of the methods from ring signature and attribute-based crypto systems, it lets several authorities distribute attribute keys independently, and granters can sign for the their identity without leaking identity or attribute information to anyone and users can flexibly select the form of their attributes to meet verification rules of the verifiers.

We prove that our scheme is EUF-CMIAA and that it provides full anonymity of both signer and attribute with respect to a random oracle model. Empirical performance evaluations validate the computational cost reduction, and reveal a 30 percentage decrease in the number of $\mathbb {G}_T$ exponentiations and scalar multiplications during signing operation with respect to the state-of-the-art SM9-based ring signature scheme, which is tailored to the resource-limited clients participating in distributed and DID-based environments.

Nevertheless, the current construction inherits linear growth in signature size and verification time relative to the number of ring members and attributes. Future work will focus on designing a constant-size attribute ring signature that maintains security guarantees while eliminating scalability limitations. Incorporating these will make our approach more feasible to employ in the big scale zero-trust system where access control is dynamic, and data is distributed.

## Data Availability

Data is provided within the manuscript.
